# Supplementary material for: The Contribution of Legionella anisa to Legionella Contamination of Water in the Built Environment
Source: Int J Environ Res Public Health. 2024 Aug 20;21(8):1101. doi: 10.3390/ijerph21081101 (PMC11354164; doi:10.3390/ijerph21081101)
Supplement: Supplementary file 1 [file ijerph-21-01101-s001.zip › ijerph-3111805-supplementary.pdf]

Supplementary Materials

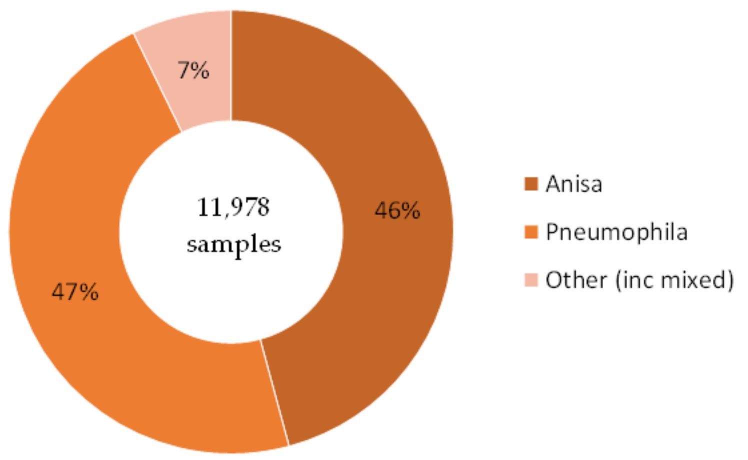

Figure S1. Data Set 1—Total number of *Legionella*-positive samples recorded, categorized by species.

Table S1. Data Set 1—Total number of *Legionella*-positive samples recorded, categorized by date and species.

Date	*L. anisa*		*L. pneumophila*		Other (incl. Mixed)		TOTAL
**July 2019**	**80**	**(58%)**	50	(36%)	7	(5%)	137
August 2019	147	(43%)	162	(47%)	33	(10%)	342
September 2019	156	(43%)	186	(52%)	19	(5%)	361
October 2019	154	(44%)	168	(48%)	27	(8%)	349
November 2019	118	(46%)	114	(44%)	25	(10%)	257
December 2019	137	(45%)	141	(46%)	28	(9%)	306
January 2020	101	(40%)	125	(50%)	26	(10%)	252
February 2020	209	(51%)	185	(45%)	16	(4%)	410
March 2020	240	(56%)	177	(41%)	14	(3%)	431
April 2020	57	(31%)	115	(63%)	11	(6%)	183
May 2020	169	(26%)	455	(70%)	26	(4%)	650
June 2020	229	(50%)	206	(45%)	27	(6%)	462
July 2020	504	(53%)	377	(39%)	76	(8%)	957
August 2020	421	(51%)	361	(43%)	48	(6%)	830
September 2020	401	(59%)	239	(35%)	34	(5%)	674
October 2020	344	(49%)	322	(46%)	30	(4%)	696
November 2020	298	(57%)	196	(38%)	28	(5%)	522
December 2020	241	(49%)	209	(43%)	38	(8%)	488
January 2021	98	(43%)	117	(51%)	13	(6%)	228
February 2021	101	(38%)	152	(58%)	11	(4%)	264
March 2021	137	(37%)	217	(58%)	21	(6%)	375
April 2021	154	(49%)	142	(45%)	21	(7%)	317
May 2021	266	(39%)	364	(54%)	47	(7%)	677
June 2021	122	(23%)	281	(52%)	138	(26%)	541
July 2021	337	(43%)	387	(49%)	59	(8%)	783
August 2021	266	(55%)	175	(36%)	45	(9%)	486
TOTAL	5,487	(46%)	562	3(47%)	868	(7%)	11,978

N.B. Row percentage in brackets

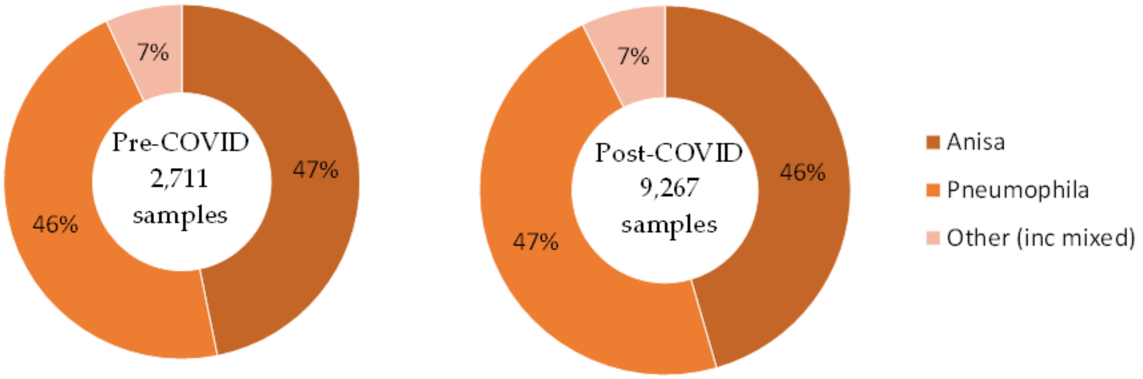

Figure S2. Data Set 1—Number of positive samples recorded before and after the COVID-19 outbreak, categorized by species.

Table S2. Data Set 2–Total number of *Legionella*-positive samples recorded, categorized by date and species, for pre- and post-cleaning.

Date	Pre-Cleaning Samples				Post-Cleaning Samples			
	Anisa	Pneumophila	Other	Total	Anisa	Pneumophila	Other	Total
Jan 2020					1	3	0	**4**
Feb 2020					9	1	3	**13**
Mar 2020					7	1	1	**9**
May 2020					5	1	2	**8**
Jun 2020	5	4	0	**9**	4	4	0	**8**
Jul 2020	4	1	7	**12**	6	0	1	**7**
Nov 2020	12	8	6	**26**	5	10	2	**17**
Dec 2020	6	9	0	**15**	2	4	0	**6**
Feb 2021	2	9	0	**11**	3	7	1	**11**
Mar 2021	13	8	3	**24**	5	13	0	**18**
Apr 2021	11	7	5	**23**	6	11	2	**19**
May 2021	4	17	0	**21**		12	2	**14**
Jun 2021	4	18	4	**26**	7	10	4	**21**
**Total**	**61**	**81**	**25**	**167**	**60**	**77**	**18**	**155**

Table S3. Data Set 2—Effect of cleaning intervention on colony counts (CFU/L) and species' predominance in showers and handwashing-basin taps with TMVs.

Pre- and post-cleaning species-predominance data[1]	Showers[2]	TMV taps[2]
No change from *L. pneumophila* Sg1 with counts reduced	17 (23%)	12 (7.4%)
No change from *L. pneumophila* Sg1 with counts increased	4 (5.4%)	5 (3.1%)
No change from *L. anisa* with counts reduced	9 (12.2%)	3 (1.9%)
No change from *L. anisa* with counts increased	4 (5.4%)	2 (1.2%)
Change from *L. pneumophila* Sg1 to *L. anisa* with counts reduced	1 (1.4%)	4 (2.5%)
Change from *L. pneumophila* Sg1 to *L. anisa* with counts increased	0	2 (1.2%)
Change from *L. anisa* to *L. pneumophila* Sg1 with counts reduced	0	1 (0.6%)
Change from *L. anisa* to *L. pneumophila* Sg1 with counts increased	0	4 (2.5%)
Change from *L. pneumophila* Sg1 to none detected	1 (1.4%)	13(8.1%)
Change from none detected to *L. pneumophila* Sg1	0	4 (2.5%)
Change from *L. anisa* to none detected	11 (14.9%)	21 (13%)
Change from none detected to *L. anisa*	3 (4.1%)	3 (1.9%)
No change from none detected	11 (14.9%)	57 (35.4%)
Others (*L. pneumophila* Sg2-15; *Legionella* spp.; mixed cultures)	13	30
**Total**	**74**	**161**

1Predominant Legionella species isolated from sampling points (showers or TMV taps), and whether colony counts of predominant species were increased, showed no change, or decreased post-cleaning compared to pre-cleaning.
2n = number of sample points.
